# Report on the Current Inventory of the Toolbox for Plant Cell Wall Analysis: Proteinaceous and Small Molecular Probes

**DOI:** 10.3389/fpls.2018.00581

**Published:** 2018-05-03

**Authors:** Maja G. Rydahl, Aleksander R. Hansen, Stjepan K. Kračun, Jozef Mravec

**Affiliations:** ^1^Department of Plant and Environmental Sciences, University of Copenhagen, Frederiksberg, Denmark; ^2^GlycoSpot IVS, Frederiksberg, Denmark

**Keywords:** cell wall, polysaccharide, molecular probe, monoclonal antibody, carbohydrate-binding module, metabolic labeling, glycan microarray, imaging

## Abstract

Plant cell walls are highly complex structures composed of diverse classes of polysaccharides, proteoglycans, and polyphenolics, which have numerous roles throughout the life of a plant. Significant research efforts aim to understand the biology of this cellular organelle and to facilitate cell-wall-based industrial applications. To accomplish this, researchers need to be provided with a variety of sensitive and specific detection methods for separate cell wall components, and their various molecular characteristics *in vitro* as well as *in situ*. Cell wall component-directed molecular detection probes (in short: cell wall probes, CWPs) are an essential asset to the plant glycobiology toolbox. To date, a relatively large set of CWPs has been produced—mainly consisting of monoclonal antibodies, carbohydrate-binding modules, synthetic antibodies produced by phage display, and small molecular probes. In this review, we summarize the state-of-the-art knowledge about these CWPs; their classification and their advantages and disadvantages in different applications. In particular, we elaborate on the recent advances in non-conventional approaches to the generation of novel CWPs, and identify the remaining gaps in terms of target recognition. This report also highlights the addition of new “compartments” to the probing toolbox, which is filled with novel chemical biology tools, such as metabolic labeling reagents and oligosaccharide conjugates. In the end, we also forecast future developments in this dynamic field.

## Introduction to tools for probing cell walls

Plant cell walls play an important role in numerous cellular functions and developmental processes including the mediation of cell-to-cell adhesion, provision of mechanical support, and interaction with pathogens and the environment (Albersheim et al., 2010; Wolf et al., 2012; Malinovsky et al., 2014; Chebli and Geitmann, 2017; Höfte and Voxeur, 2017). Cell walls can contribute up to 90% of the dry matter of a plant and are a crucial component of nature-derived industrial feedstock. This is becoming increasingly important for sustainable energy and novel biomaterials (Marriott et al., 2016). Due to the requirement for their multi-functionality, cell walls are complex and heterogeneous structures with a high level of spatiotemporal dynamics (Drakakaki, 2015; Barnes and Anderson, 2017; Höfte and Voxeur, 2017). In addition, cell wall composition and architecture varies between different cell types, developmental stages (Wilson et al., 2015; Monniaux and Hay, 2016; Mravec et al., 2017a), and also between species (Sørensen et al., 2011; Fangel et al., 2012; Popper et al., 2014; Leroux et al., 2015; Harholt et al., 2016). The cell wall is, for the most part, built from polysaccharides (cellulose, hemicelluloses, and pectins), proteoglycans (extensins and arabinogalactan proteins), and, in some cells, polyphenolics (lignin) and polyesters (cutin and suberin). Each of these components has its own particular function(s) within the intricate cell wall structure (Albersheim et al., 2010; Burton et al., 2010). However, scientists still face many open-ended questions regarding the biology of plant cell walls, particularly with respect to the compartmentalized synthesis of cell wall components, *in muro* dynamic co-ordination of the cell wall architecture, the relationship between different cell wall components, and the activity of regulatory loops coupled to intrinsic cellular, developmental and pathogenesis-related pathways.

The diverse techniques used to study cell walls relies heavily on detection probes that are specific for cell wall components (**C**ell **W**all **P**robes-CWPs). In this review, a CWP will be defined as any molecule with the ability to specifically bind (or be incorporated into) a cell wall component, therefore allowing subsequent selective detection, quantification or visualization. Here it is important to note that “chemical probe,” in the field of chemical biology, is a wider term that can also be used to describe molecules that are able to influence the function of the target, e.g., by altering its activity via specific binding (Garbaccio and Parmee, 2016).

**Figure 1:**
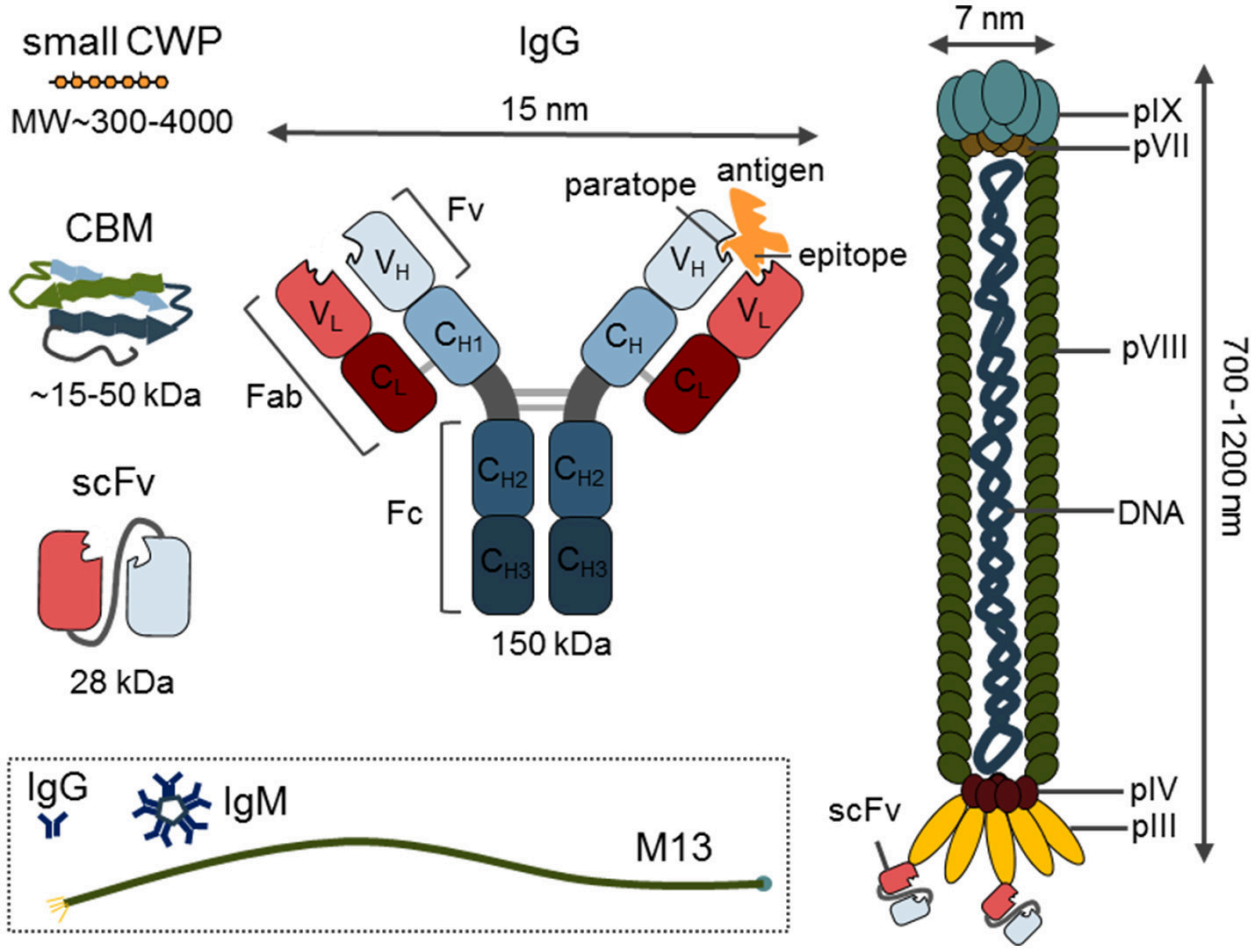
Overview of the different classes of probes with a size comparison. Depicted are small cell wall probe (CWP), carbohydrate-binding module (CBM), single chain variable fragment (scFV) and immunoglobulin G (IgG) structure with indicated regions and size in kDa. The structure of M13 phage with indicated capsid structural proteins (pIII, pIV, pVII, pVIII, pIX). The content of the dashed square indicates the real-size relation of immunoglobulins IgG and IgM to the M13 phage.

Although many biochemical and biophysical techniques, such as mass spectroscopy (MS) or nuclear magnetic resonance (NMR), can accurately decipher the detailed structure of cell wall components, they are not high-throughput (HTP) and usually require homogenization of samples, harsh pre-treatments or extractions, and a high-level of expertise from the operating personnel. By contrast, CWPs can be used to detect various cell wall components (specifically, their relative abundance and structural alterations) in a HTP manner, whilst simultaneously providing insights into their molecular structure and interactions with other glycans. In addition, this understanding of the cell wall material can often be acquired by detecting polymers in their native context without having to deconstruct the cell wall. As such, CWPs are vital for the study of different aspects of cell wall microstructure *in muro* (Knox, 2008; Lee et al., 2011), and can be used to follow the dynamics of the cell wall, and its components, *in planta* (Wallace and Anderson, 2012; Voiniciuc et al., 2018). The current set of available CWPs (Figure 1) consists of anti-glycan monoclonal antibodies (mAbs), phage display-based probes, carbohydrate-binding modules (CBMs), and small molecular probes such as fluorophores, oligosaccharide conjugates, and building blocks for metabolic labeling. In the following sections we will review the developments in this field, and discuss technical aspects and the important features of various classes of CWPs.

## Proteinaceous probes—combining diversity with versatility

### Antibodies—the largest compartment of the CWP tool box

**Figure 2:**
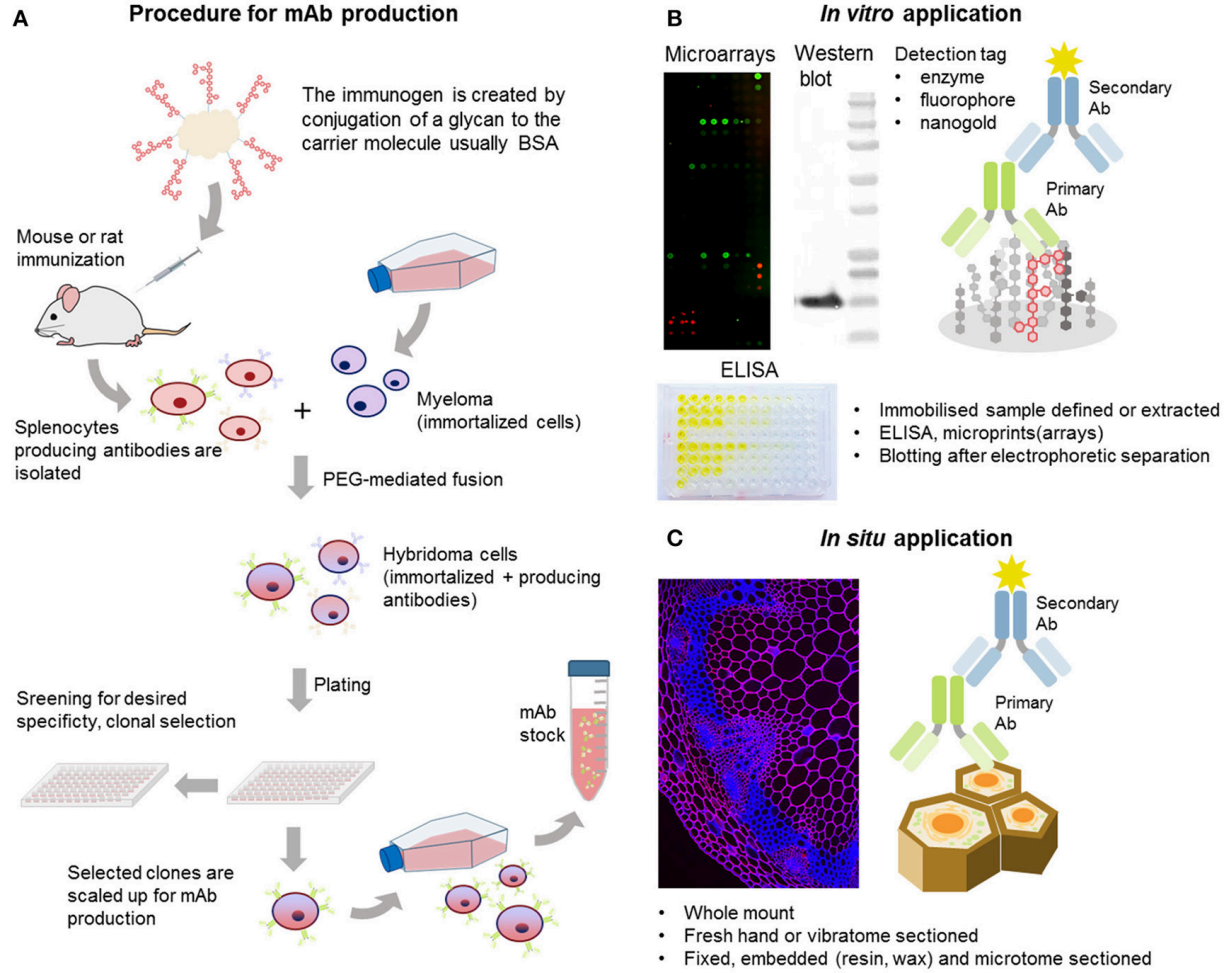
Generation and examples of mAb applications. **(A)** Simplified scheme of the standard procedure for anti-glycan mAb production. Unlike whole proteins, glycans are conjugated to a carrier molecule (typically BSA or KLH) before immunization. **(B)** Generated mAbs can be used *in vitro* to profile cell wall extracts in microarray applications or in ELISA-based methods like epitope detection chromatography providing also more structural information. **(C)** MAbs can be also used *in situ* for localization of the target molecules *in situ* on various types of plant material whole mount or sectioned by various methods.

Antibodies are the largest, and possibly the most important, component of the CWP tool box. They are highly specific, versatile, and can be applied to a diverse range of techniques (Cummings et al., 2017; Figure 2). One of the biggest advantages of antibodies is that they can be developed against virtually any antigen, as long as there is a significant immune response in the host. The production of antibodies against plant carbohydrates was initiated in the 1980's and, to date, hundreds of plant cell wall-related antibodies have been reported, covering all classes of the major polymeric components and some of their molecular characteristics (Pattathil et al., 2012). Tables 1–5 provide an overview of the most notable antibodies related to pectins (Table 1), hemicelluloses (Table 2), proteoglycans (Table 3), cell wall phenolics (Table 4), and algal polysaccharides (Table 5). The website^1^ curated by the CCRC, at the University of Georgia, is another great resource for mining information regarding cell wall related antibodies, including their specificities.

**Table 1:** A selection of commonly used antibodies related to pectins.

	**Ab**	**Epitope**	**Source**	**References**
Homogalacturonan	JIM5	HG with a low DE (range of partially methyl-esterified/unesterified HG)	Rat	Knox et al., 1990; Willats et al., 2000; Clausen et al., 2003; Verhertbruggen et al., 2009a
	JIM7	HG with a high DE	Rat	Knox et al., 1990; Willats et al., 2000; Clausen et al., 2003; Verhertbruggen et al., 2009a
	LM18	HG, partially methylesterified or unesterified	Rat	Verhertbruggen et al., 2009a
	LM19	HG, preferably unesterified (more selective than JIM5)	Rat	Verhertbruggen et al., 2009a
	LM20	HG with a high DE (more selective than JIM7)	Rat	Verhertbruggen et al., 2009a
	CCRC-M130/M34	HG with a high DE (cluster with JIM7)	Mouse	Pattathil et al., 2010
	CCRC-M38	Unesterified HG; DP>5	Mouse	Pattathil et al., 2010
	2F4	Calcium crosslinked HG (egg boxes)	Mouse	Liners et al., 1989
	LM8	Xylogalacturonan	Rat	Willats et al., 2004
	LM7	Partially methylesterified HG (non-blockwise de-esterification processes) also alginates	Rat	Clausen et al., 2003; Torode et al., 2015
	PAM1	Long stretches (over 30 units) of unesterified HG	phage display (scFv)	Willats et al., 1999, 2000; Manfield et al., 2005
RG-I	CCRC-M60	Rhamnogalacturonan I and AGP	Mouse	Pattathil et al., 2010
	INRA-RU1/RU2	Backbone of rhamnogalacturonan I	Mouse	Ralet et al., 2010; Ruprecht et al., 2017
	CCRC-M14/M35/M36/M69/M129	Backbone of rhamnogalacturonan I	Mouse	Ruprecht et al., 2017
	CCRC-M2	Rhamnogalacturonan Ia	Mouse	Pattathil et al., 2010
	LM5	(1 → 4)-β-D-galactan with at least three galactose units at non-reducing end.	Rat	Jones et al., 1997; Andersen et al., 2016; Torode et al., 2017
	XD3	(1 → 4)-β-D-galactan	Phage (scFv)	Shinohara et al., 2015
	CCRC-M7	6-linked β-D-Gal oligomers that contain arabinose	Mouse	Pattathil et al., 2010
	LM6	(1 → 5)-α-L-arabinan / AGP epitopes	Rat	Willats et al., 1998; Verhertbruggen et al., 2009b
	LM13	Specific subset of unbranched pectic (1 → 5)-α-L-arabinan (arabinanase sensitive)	Rat	Moller et al., 2008; Verhertbruggen et al., 2009b
	LM16	Processed α- (1 → 5)-α-L-arabinan. Epitope might be part of galactosyl residue(s) on RG backbones (galactosidase sensitive)	Rat	Verhertbruggen et al., 2009b
	LM26	(1 → 4)- β-D-galactan substituted with β-(1 → 6) galactosyl, three galactose residues required as backbone.	Rat	Torode et al., 2017
	INRA-AGI-1	RGI related linear chain of (1 → 4)-linked Gal and (1 → 5)-linked Ara	Mouse	Buffetto et al., 2015
	CCRC-M11/M12/M15	Arabinogalactan epitope on RG-I and AGP	Mouse	Pattathil et al., 2010
RG-II	RG-II	Rhamnogalacturonan II	Rabbit (pAb)	Matoh et al., 1998
	CCRC-R1	Rhamnogalacturonan II (unesterified)	Phage (Fab)	Williams et al., 1996

**Table 2:** A selection of commonly used antibodies related to hemicelluloses.

	**Ab**	**Epitope**	**Source**	**References**
Mannans	BS-400-4 (BGM C6)	(1 → 4)-β-D-(galacto)mannan	Mouse	Pettolino et al., 2001
	LM21	(1 → 4)-β-D-(galacto)(gluco)mannan; DP2 to DP5	Rat	Marcus et al., 2010
	LM22	(1 → 4)-β-D-(gluco)mannan; DP2 to DP5	Rat	Marcus et al., 2010
	CCRC-M70	Galactomannan	Mouse	Pattathil et al., 2010
	CCRC-M169	Acetylated mannan	Mouse	Pattathil et al., 2012; Zhang et al., 2014
	CCRC-M170	Acetylated glucomannan	Mouse	Pattathil et al., 2012; Zhang et al., 2014
Beta glucans	BS-400-2 (LAMP2H12H7)	(1 → 3)-β-D-glucan (callose and laminarin)	Mouse	Meikle et al., 1991
	BS-400-3 (BG1)	(1 → 3), (1 → 4)-β-D-glucan (MLG)	Mouse	Meikle et al., 1994
	LM15	Xyloglucan (XXXG motif), non-fucosylated (can accommodate a single Gal residue) Requires a single unsubstituted Glc on the non-reducing end	Rat	Marcus et al., 2008; Ruprecht et al., 2017
	LM24	Xyloglucan (XLLG motif)	Rat	Pedersen et al., 2012
	LM25	Xyloglucan	Rat	Pedersen et al., 2012
	CCRC-M1	α-L-fucosylated xyloglucan (also RG-I)	Mouse	Puhlmann et al., 1994; Pattathil et al., 2010
	CCRC-M86/100/103	Internal xyloglucan chain, requires non-substituted Glc residue toward non-reducing end.	Mouse	Ruprecht et al., 2017
	CCRC-M93/95/96/101/104	Xyloglucan with Gal substitution	Mouse	Dallabernardina et al., 2017
Xylans	LM10	(1 → 4)-β-D-xylan	Rat	McCartney et al., 2005; Ruprecht et al., 2017
	LM11/CCRC-M147/149	(1 → 4)-β-D-xylan/arabinoxylan, high tolerance of backbone substitutions	Rat	McCartney et al., 2005; Ruprecht et al., 2017
	CCRC-M140/160/137/139/152	(1 → 4)-β-D-xylan, low tolerance for substitutions	Rat	Ruprecht et al., 2017
	CCRC-M108/109/110	(1 → 4)-β-D-xylan substituted with Ara on the 2-position.	Mouse	McCartney et al., 2005; Ruprecht et al., 2017
	LM23	Non-acetylated xylosyl residues, pectic xylogalacturonan and xylan	Rat	Ruprecht et al., 2017
	LM27	Grass glucuronoarabinoxylan (GAX)	Rat	Cornuault et al., 2015
	LM28	(1 → 4)-β-D-xylan with GlcA substitution on 2-position, both methyl or non-methylesterified	Rat	Cornuault et al., 2015
	INRA-AX1	Backbone of xylans	Mouse	Guillon et al., 2004
	INRA-UX1	Alkali treated glucuronoxylan	Mouse	Koutaniemi et al., 2012
	CCRC-M150	(1 → 4)-β-D-xylan with GlcA substitution which is not methylesterified	Mouse	Ruprecht et al., 2017
	CCRC-M144/145/146/155	(1 → 4)-β-D-xylan with GlcA substitution which is methylesterified at 4-*O*-position	Mouse	Ruprecht et al., 2017

**Table 3:** A selection of antibodies related to proteoglycans.

	**Ab**	**Epitope**	**Source**	**References**
Extensins	LM1	Extensins and hydroxyproline-rich glycoproteins (HRGP)	Rat	Smallwood et al., 1995
	JIM11	Extensins, periodate sensitive epitope	Rat	Smallwood et al., 1994
	JIM12	Extensins, epitope includes a protein component (proteinase sensitive)	Rat	Smallwood et al., 1994;
	JIM20	Extensins, periodate sensitive epitope	Rat	Smallwood et al., 1994;
	JIM19	Extensins, periodate sensitive epitope	Rat	Knox et al., 1995, Wang et al., 1995
Arabinogalactan and AGP	LM2	AGP, (1 → 6)-β-D galactan chain with terminally attached GlcA.	Rat	Yates et al., 1996; Ruprecht et al., 2017
	LM14	Arabinogalactan and AGP	Rat	Moller et al., 2007
	JIM4	AGP (β-D-GlcA-(1 → 3)-α-D-GalA-(1 → 2)-α-D-Rha competes for binding)	Rat	Knox et al., 1989; Yates et al., 1996; Ruprecht et al., 2017
	JIM13	AGP, periodate sensitive epitope	Rat	Knox et al., 1991; Yates et al., 1996
	JIM14	AGP, unsubstituted (1 → 6)-β-D galactan chain	Rat	Knox et al., 1991; Yates et al., 1996; Ruprecht et al., 2017
	JIM16	AGP, (1 → 3)-β-D galactan chain when substituted with a single β-D- (1 → 6)-linked Gal residue	Rat	Knox et al., 1991; Yates et al., 1996; Ruprecht et al., 2017
	PN 16.1B3	AGP	Mouse	Norman et al., 1986
	MH4.3E5	Arabinogalactan and AGPs	Mouse	Hahn et al., 1987
	MAC207	AGP from pea (β-D-GlcA-(1 → 3)-α-D-GalA-(1 → 2)-α-D-Rha competes for binding)	Rat	Yates et al., 1996
	XD27	AGP	Phage (scFv)	Shinohara et al., 2015
	CCRC-M133	Arabinogalactan (cluster 2), (1 → 4)-β-D-galactan with DP ≥6	Mouse	Pattathil et al., 2010; Ruprecht et al., 2017
	CCRC-M85	Arabinogalactan (cluster 3)	Mouse	Pattathil et al., 2010
	CCRC-M78	Arabinogalactan (cluster 4)	Mouse	Pattathil et al., 2010

**Table 4:** A selection of antibodies related to cell wall phenolics.

**Ab**	**Epitope**	**Source**	**References**
LM12	Feruloylate/ferulic acid on any polymer and heteroxylan	Rat	Pedersen et al., 2012
LM9	Feruloylated (1 → 4)-β-D-galactan	Rat	Clausen et al., 2004
INRA-COU1	Free p-coumaric acids and coumarate esters	Mouse	Tranquet et al., 2009
INRA-COU2	Esterified p-coumaric acids	Mouse	Tranquet et al., 2009
Anti-H	Raised against synthetic lignin with H unit	Rabbit (pAb)	Ruel et al., 1994; Joseleau and Ruel, 1997
Anti-G	Raised against synthetic lignin with G unit	Rabbit (pAb)	Ruel et al., 1994; Joseleau and Ruel, 1997
Anti-GS	Raised against synthetic lignin with GS unit	Rabbit (pAb)	Ruel et al., 1994; Joseleau and Ruel, 1997
Anti-S	Raised against syringyl polymer	Rabbit (pAb)	Joseleau et al., 2004
KM1	Lignin (dehydrodiconiferyl alcohol, 8-5′ linkage)	Mouse	Kiyoto et al., 2013
KM2	Lignin (pinoresinol, 8-8′ linkage)	Mouse	Kiyoto et al., 2013

**Table 5:** A selection of antibodies related to algal polysaccharides and starch.

**Ab**	**Epitope**	**Source**	**References**
B3	Carrageenan (preference for ι-carrageenan chains in a helical conformation)	Phage (scFv)	Liners et al., 2005
BAM1-BAM4	Fucoidans (different levels of sulfation)	Rat	Torode et al., 2015
BAM6-BAM11	Alginates (different rations of mannuronic and guluronic acid)	Rat	Torode et al., 2016
INCH2	Ulvan, epitope contain the ulvanobiuronic acid 3-sulfate B structure, sensitive to ulvan lyase	Mouse	Rydahl et al., 2017
INCH1	Starch, α-(1 → 4)-linked glucan chains; DP>4	Mouse	Rydahl et al., 2017

The majority of these are mAbs produced from rat or mouse hybridoma cells, resulting from cell-based isolation procedures (Köhler and Milstein, 1975; Knox, 2008). However, a handful have also been generated as polyclonal Abs (pAbs). The standard procedure for the generation of an anti-glycan mAb is depicted in Figure 2A, where the immunogen is generated through conjugation of the target molecule to a carrier, which is usually bovine serum albumin (BSA) or keyhole limpet hemocyanin (KLH). The protocols used are not significantly different from those for the generation of anti-protein or anti-peptide mAbs. Hybridoma, as immortalized cells, can in theory be cultivated indefinitely, thus supplying high affinity mAbs in relatively large quantities. However, the loss of production capability or specificity in some hybridoma lines over time is a serious problem. To overcome these problems, switching to recombinant mAb production is recommended. In addition, new quality standards for the characterization, propagation, and tracking of cell lines are being implemented in the immuno-biotechnology field (Weller, 2016).

**Figure 3:**
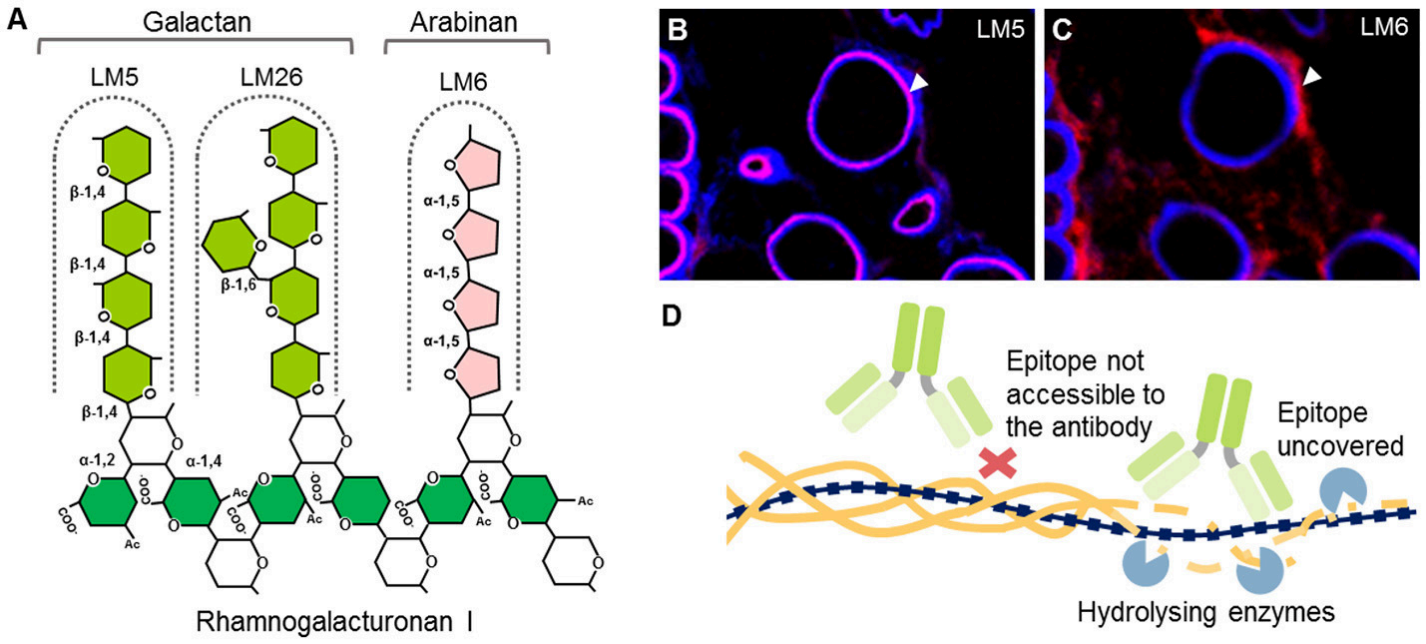
Example of mAb-recognized epitopes, cell wall heterogeneity, and masking. **(A)** Example of different epitopes recognized by mAbs on RG-I side chains. Both LM5 and LM26 mAbs bind to (1 → 4)-β-D galactan epitopes with a difference in requirement of a Gal substitution via α-D-(1 → 6) bond in the case of LM26. LM6 requires a linear chain of four α-(1 → 5) linked L-arabinose units. This epitope can be found also on AGPs. **(A,B)** LM5 and LM6 mAbs can have affinity toward different cell wall microdomains. An example of an *in situ* labeling of sections of resin embedded pea border cells with LM5 and LM6. Calcofluor White (blue channel) and signal from the secondary anti-rat antibody conjugated to Alexa Fluor 555 (red). **(B)** LM5 labels cell walls of released border cells, whereas **(C)** LM6 labels the shed cell wall material released to the environment (arrowheads). For the original experiments see Mravec et al. (2017a). **(D)** Phenomenon of masking of mAbs epitopes. Cell wall carbohydrates are arranged in tight arrays and this could prevent binding of mAbs. Pre-digestion with specific enzymes can reveal these “hidden” epitopes.

Unlike polyclonal sera, mAbs only bind one epitope (a recognized motif on the antigen molecule), a feature that is especially important in plant glycobiology. Many polysaccharides are not homogeneous polymers and instead exhibit various molecular alterations to their backbone. A classic example of this is homogalacturonan (HG), which can have different levels and patterns of methyl-esterification (Willats et al., 2001). The current set of anti-HG antibodies available can distinguish between these patterns (Verhertbruggen et al., 2009a) and some can even recognize HG in its supramolecular conformation (Table 1). For example, mAb 2F4 recognizes HG with a low DE but when it is in a complex with a divalent calcium (e.g., egg boxes, pectin gel) (Liners et al., 1989). The epitope of a carbohydrate-recognizing antibody varies in size depending on the mAb, from a single monosaccharide moiety up to a complex glycan or a long stretch of more than 10 monomeric units. Most mAbs used in the cell wall field recognize molecular structures consisting of 2–6 monosaccharide units, which is enough for them to distinguish between different types of polysaccharides. For mAbs that recognize cell wall proteoglycans, the epitope may contain a protein, a glycan part or a combination of both. See Figure 3A for an example of epitopes that can appear on one type of polysaccharide, in this case rhamnogalacturonan I (RG-I). It is important to note that some epitopes can be present on different classes of cell wall components, for example arabinogalactan chains, which are typical for both RG-I and arabinogalactan proteins (AGPs) (Pattathil et al., 2010).

mAbs are relatively easy to manipulate or modulate, and can be used in various forms. For example, expressed and purified variable small fragments of native antibodies can be utilized as a version of mAbs with a significantly reduced size (~15–55 kDa as opposed to a 150 kDa full protein) (Figure 1). Depending on the end-application, the use of a secondary antibody, or protein A or G allows for different indirect detection methods. Microarray and ELISA-based applications usually allow detection through the use of a chromogenic enzymatic reaction via horse radish peroxidase (HRP) or alkaline phosphatase (AP) conjugated to the secondary antibody (Pattathil et al., 2015a,b; Kracun et al., 2017; Figure 2B). By contrast, for *in situ* immunohistochemistry, fluorescent conjugates of secondary antibodies are usually the first choice while for transmission electron microscopy (TEM), nanogold conjugates of secondary antibodies or protein A/G are required (Wilson and Bacic, 2012) (Figure 2C). A curious fact is that the names of most mAbs are based on the names of the institutes where they have been generated: JIM, John Innes Centre Monoclonal; LM, University of Leeds Monoclonal; INRA, Institut National de la Recherche Agronomique; CCRC, Complex Carbohydrate Research Center; BS, Biosupplies; KM- Kyoto University and, finally, INCh, a collaboration between INRA and the University of Copenhagen.

### Recent additions to the repertoire of mAbs

Several new additions to the repertoire of mAbs that recognize land plant polysaccharide structures have been reported over the last 5 years, such as the INRA-AGI-1 mAb, which recognizes the RGI-related arabinogalactan linear chain (Buffetto et al., 2015), and the new xylan-related mAbs LM27 and LM28 (Cornuault et al., 2015). Lignin is a highly heterogeneous phenolic polymer, which is of great scientific interest as it has a strong influence on the effectiveness of bioconversion of plant cell walls to biofuels (Li et al., 2016). However, the number of lignin immunological probes with well-defined specificities has been limited (Table 4). In 2013, it was reported that the KM1 and KM2 antibodies could recognize two distinct phenolic linkages present in lignin (Kiyoto et al., 2013), therefore representing an important, but hitherto relatively overlooked, addition to CWPs related to cell wall components of land plants.

At present, there is an appreciable shift of interest into research on algal cell walls. Increasing our knowledge about them is essential with respect to our basic understanding of the evolution of cell walls (Popper et al., 2014; Harholt et al., 2016), and the biology of algae as a whole. Algal cell wall polysaccharides are also becoming increasingly important as pharmaceuticals, nutraceuticals and food additives (Wells et al., 2016). Recently, two sets of mAb designated as Brown Alga Monoclonal (BAM) antibodies have been developed; one against fucoidans (Torode et al., 2015) and one set against alginates (Torode et al., 2016). The latest addition to the algal probe repertoire is a mAb against the green algae polysaccharide ulvan (Rydahl et al., 2017; Table 5). However, algal polysaccharides are highly diverse molecules and there are still many potential targets. For instance, no antibodies have been reported against red algae polysaccharides agarose and its derivative porphyran. The current set of mAbs should still be extended with those having well-defined specificities against special structural conformations of algal polysaccharides, substitutions on the backbone and other occurring variable molecular features. Recent advances in HTP screening platforms reviewed in the following paragraph could be highly beneficial in generating such CWPs.

### Detailed characterization of recognized epitopes by chemical synthesis and HTP screening

One of the most challenging aspects of mAb development is the precise determination of the recognized epitopes. Large series of mAbs have been generated in the past but the descriptions of their binding patterns were often vague. This led to the development of HTP screening platforms of mAbs. Over the last 10 years, they have been already used for extensive hierarchical clustering analysis of antibody specificities (Moller et al., 2008; Pattathil et al., 2010), and in combination with chemical synthesis of pure oligosaccharides, have enabled a more detailed characterization of epitope structures (Moller et al., 2008; Pedersen et al., 2012; Andersen et al., 2016; Dallabernardina et al., 2017; Ruprecht et al., 2017; Torode et al., 2017). Notably, a large set of CCRC antibodies have recently been characterized in detail using 88 artificial oligosaccharides related to xylan, xyloglucan, RG-I, and arabinogalactan chains, and some patterns were resolved in high resolution (Ruprecht et al., 2017). Another recent study more deeply deciphered the epitopes of two galactan-specific mAbs: LM5 and LM26. This study showed that LM5 requires at least three galactose units at the non-reducing end of the galactan chain (Andersen et al., 2016) while LM26 requires a single substitution with a Gal residue via a β-(1 → 6) bond and at least three units of the backbone (Torode et al., 2017; Figure 3A). Both antibodies also exhibit interesting complementary labeling patterns in phloem (Torode et al., 2017) that relates to the mechanical properties of the cells in this tissue. Similarly, a group of xyloglucan-specific mAbs requiring Gal substitutions were characterized by Dallabernardina et al. (2017). We expect that these valuable efforts, which show the great potential of HTP platform in characterizing mAbs, will continue in providing a refined specificity data set for most mAbs.

### Remaining gaps in mAb availability

Despite the existence of a large panel of mAbs, there are still significant gaps with respect to target recognition. These gaps limit research efforts to understanding the biological role of some polysaccharides but also their endogenous chemical modifications. This is especially true for *O*-acetylation, a common modification that has a significant negative effect on biomass bioconversion. Although acetyl esters appear on many polymers (Gille and Pauly, 2012; Nafisi et al., 2015), so far only mAbs against acetylated mannan have been reported (CCRCM169 and CCRC-M170) (Pattathil et al., 2012; Zhang et al., 2014).

Interestingly, no antibody has yet been generated toward the most abundant polymer in nature—cellulose. CBM3a is currently used but lacks a high-degree of specificity toward cellulose (Blake et al., 2006; Hernandez-Gomez et al., 2015). The development of highly specific mAbs as tools truly capable of discriminating between amorphous and crystalline cellulose would be a major breakthrough, especially for the analysis of secondary cell wall formation, or for monitoring of biomass deconstruction and the saccharification process in its entirety. Hopefully, the recent success in the generation of anti-starch mAbs (Rydahl et al., 2017) might invoke a necessary momentum and optimism for the initiation of such efforts.

Polyesters, like cutin or suberin, are also deposited in the extracellular space of some specialized tissues where they play an essential role in water management and the formation of a semipermeable barrier between the plant and environment (Andersen et al., 2015; Fich et al., 2016). These compounds are constructed from various monomeric units and present in highly localized cell wall microdomains, such as the Casparian strip (Andersen et al., 2015). Currently, their visualization *in situ* relies solely on cytological staining. If developed in the future, CWPs for these special cell wall components and their numerous structural variants, could be used in conjunction with immunolocalization of relevant biosynthetic enzymes. This can help to unravel how these compounds are synthesized and spatially deposited in a very site-restrictive manner.

The lack of specific detection tools is not only limited to above mentioned cell wall components but also to their fine *in muro* arrangement. Cell walls are constructed as an intricate array where different polysaccharides are mutually interlinked to form desired spatial 3D configurations. These linkages can form through covalent bonding (Tan et al., 2013; Cornuault et al., 2015) or non-covalent association (e.g., hydrogen bonding) between various classes of polymers (Cosgrove, 2016). Visualization and quantification of these intermolecular linkages with probes would dramatically advance the study of cell walls and allow a deeper understanding their dynamics during development. This is especially true for the cell wall remodeling during cell elongation that involves rapid dissociation of xyloglucan and cellulose microfibrils after apoplastic acidification (Cosgrove, 2016; Höfte and Voxeur, 2017). To visualize this elusive biological phenomenon in real-time using CWPs, would require highly unorthodox approaches, some of which are discussed later in this review.

### Non-conventional approaches in the generation of novel mAbs

Antibody development is often limited because some molecules do not induce an adaptive immune response in the immune system of the host animal. The level of immunogenicity of plant polysaccharides is known to be much lower than for proteins, partially due to the fact that their 3D molecular structure is not as complex as those of proteins. In addition, different pathways are induced in response to carbohydrate and protein structures (Cunto-Amesty et al., 2001). The lack of a sufficient immune response to certain carbohydrate moieties can, in some cases, be explained by the fact that some of these molecules are a part of an animal's daily feed. Low immunogenicity is a well-known, although relatively surprising, problem in the case of rhamnogalacturonan II (RG-II) despite its highly complex molecular structure (Albersheim et al., 2010; Pabst et al., 2013). Although, polyclonal antibodies specific to RG-II have been produced (Matoh et al., 1998), no reliable anti-RG-II mAbs have been reported thus far. It has been speculated that this is due to the high-degree of variation in its structure and the level of methylation in different subdomains (Pabst et al., 2013).

One way to circumvent these obstacles could be to develop antibodies in a more randomized manner, where instead of using a single well-defined antigen, a complex mixture of antigens (e.g., whole cell wall extracts) is used in a form of “shotgun” immunization. An antigen overload, or presentation of the antigen in the context of the whole cell wall glycome, might hijack the host's immune response (Rydahl et al., 2017). This has recently been proven to be a successful way to overcome the barrier of the limited immunogenicity of starch (Rydahl et al., 2017). Such an approach could also be a way to remove the existing bias toward well-known carbohydrates, which are found in the primary and secondary cell walls of higher plants. Immunization using crude non-separated samples from e.g., algae, living fossils, less studied or rare species, followed by simultaneous characterization of the mAbs generated and the antigens by HTP analytical techniques may lead to the identification of completely new polysaccharides, or other cell wall components, by having probes for them already in hand.

### *In vitro* applications of mAbs

One of the most powerful applications of anti-glycan mAbs is in HTP glycan profiling, either in nitrocellulose-based microarrays (also called comprehensive microarray polymer profiling, CoMPP) or ELISA-based methods (Moller et al., 2007; Pattathil et al., 2015a; Kracun et al., 2017). Since its introduction, HTP cell wall profiling has proved to be extremely beneficial in the characterization of the cell walls of different species (Sørensen et al., 2011; Fangel et al., 2012; Hervé et al., 2016), elucidation of the properties and composition of biomass (Pattathil et al., 2015b; Djajadi et al., 2017), analysis of the tissue specific distribution of given epitopes (Leroux et al., 2015; Wilson et al., 2015), and for studying enzymatic characteristics (Vidal-Melgosa et al., 2015; Walker et al., 2017). During CoMPP, the extracted material is printed in the form of a microarray using a nitrocellulose matrix. In the case of ELISA-based assays, the extractions are immobilized on an immunosorbent plate and probed. Glycan profiling can be done in tandem with other biochemical methods thus expanding the data obtained such as for example, more structural information. This includes, for instance, epitope detection chromatography (EDC), which couples size-exclusion or anion-exchange chromatography and immunodetection (Cornuault et al., 2014), or HTP analysis of enzymes with cell wall degrading activities using microarray printing and profiling of the reaction products (Vidal-Melgosa et al., 2015).

One of the major limitations of these applications is the semi-quantitative nature of mAb probing. The data produced are usually presented as normalized relative values but they do little to account for the different avidity and affinity of the various mAbs. This can lead to the risk of misinterpretation of results. Affinity is the strength of the interaction between an epitope and its corresponding paratope, while avidity is the total strength of the interaction between the antigen and the entire immunoglobulin and is dependent on its valence. Two antibodies can have different affinities and avidities even when recognizing the same epitope. In other words, value signals obtained for two different antibodies do not necessarily reflect the actual difference in epitope abundance. Moreover, the quantification of an antigen's absolute concentration by comparison to known standards is hindered by the non-linear nature of color product development in e.g., HRP or AP-based secondary probing. Taken together, caution should be exercised when comparing different antibodies, as well as when different detection methods are used for the same antibody.

### *In situ* applications of mAbs

mAbs are also usually a researcher's first choice for use in *in situ* analysis (Hervé et al., 2011; Avci et al., 2012; Verhertbruggen et al., 2017). mAbs can be used either with whole mounts, for instance, for surface labeling of *Arabidopsis* roots (Larson et al., 2014) or pollen tubes (Chebli et al., 2012) on fresh hand- or vibratome-sectioned material, or on embedded and (ultra-, cryo-) microtome sectioned material. Figures 3B,C shows resin-embedded and sectioned pea root apices that have been probed with two mAbs (LM5 and LM6) as an example of *in situ* analysis of the heterogeneity of cell walls. There are considerable methodological differences between the various methods for sectioning and their applications to address particular scientific questions. These have been extensively compared by Verhertbruggen et al. (2017). The large size of mAbs is sometimes an obstacle for accurate analysis, even in very thinly sectioned material. A phenomenon described as “masking” can affect the results of *in situ* localization studies (Marcus et al., 2008). Cell wall polysaccharides are arranged in a very compact way in the cell wall, interacting though weak interactions (hydrogen bonds, van der Waals interactions), which limits the accessibility of the antibodies to the epitopes (Figure 3D). However, pre-treatment of samples with various hydrolytic enzymes can reveal, or “unmask,” the hidden epitopes (Marcus et al., 2008, 2010; Xue et al., 2013; Kozlova et al., 2014; Buffetto et al., 2015). Although masking is often considered to be an experimental hurdle, a detailed elucidation of this phenomenon may provide important, and otherwise elusive, information about the nature of tight arrays of polysaccharides and their mutual interaction *in muro*. mAbs are also less efficient than other smaller probes (discussed below) for *in planta* studies, not only because of their size. For instance, mAbs are produced at mammalian pH, which is much higher than the normal acidic pH of the apoplast (Cosgrove, 2005). This could limit the binding of some antibodies when used directly in growth media or generate artifacts. Furthermore, preservatives like sodium azide—present in commercial antibody preparations—may have a toxic effect on the living sample.

### Phage display—generation of cell wall binders without immunization

In the late 1980's, the expansion of molecular biology methods and DNA cloning also allowed the development of innovative ways to produce synthetic antibodies. Phage display technology is a recombinant technique for the screening and selection of recombinant paratopes against a desired compound (McCafferty et al., 1990). Unlike traditional antibody production, which uses the host system for antibody selection, this approach requires potential binders to be expressed and presented on the surface of a phage (bacterial virus; most commonly the M13 phage). A library of DNA sequences of vFc fragments are cloned into a phagemid vector and the fragment is then expressed in fusion with one of the phage capsid proteins (Figure 1, Nissim et al., 1994). Several rounds of panning steps with an immobilized antigen and *E. coli* host reinfection can allow selection of the strongest and most specific binders. In terms of advantages, this method of production can be targeted against any molecule. It is a valuable second choice when classical antibody production is unsuccessful due to a low immunological response in the host. Finally, the use of phage display has a strong bioethical advantage over classical antibody production as it does not use laboratory animals.

However, the current selection of reported phage display-based probes is not yet extensive. The CCRC-R1 phage was the first attempt to overcome the low immunogenicity of RG-II (Williams et al., 1996), and was followed by the better known and more widely used PAM1, which recognizes long stretches of HG lacking ester groups (more than 30) (Willats et al., 1999, 2000). Other phage display-generated probes include B3 (anti-carrageenan) (Liners et al., 2005), XD3 [anti-(1 → 4)-β-D-galactan], and XD27 (anti-AGP) (Shinohara et al., 2015). One of the main disadvantages of the phage display approach is that the panning selection requires the binding of whole phage particles (see Figure 1 dashed square for size comparison to mAbs) and, therefore, strongly relies on the strength of the paratope-epitope interaction. Experience shows that charged molecules (e.g., sulfated polysaccharides or HG with low DE) that are able to form strong polar interactions are usually the best target for phage display. Another limitation is that only small variable fragments, like scFv or random peptide libraries, are suitable for bacterial expression in phage display. Due to the size limitations of the phagemid vector, whole antibodies cannot be presented on phages (Steinwand et al., 2014).

### CBMs—proteinaceous probes with a moderate size and high manipulability

**Figure 4:**
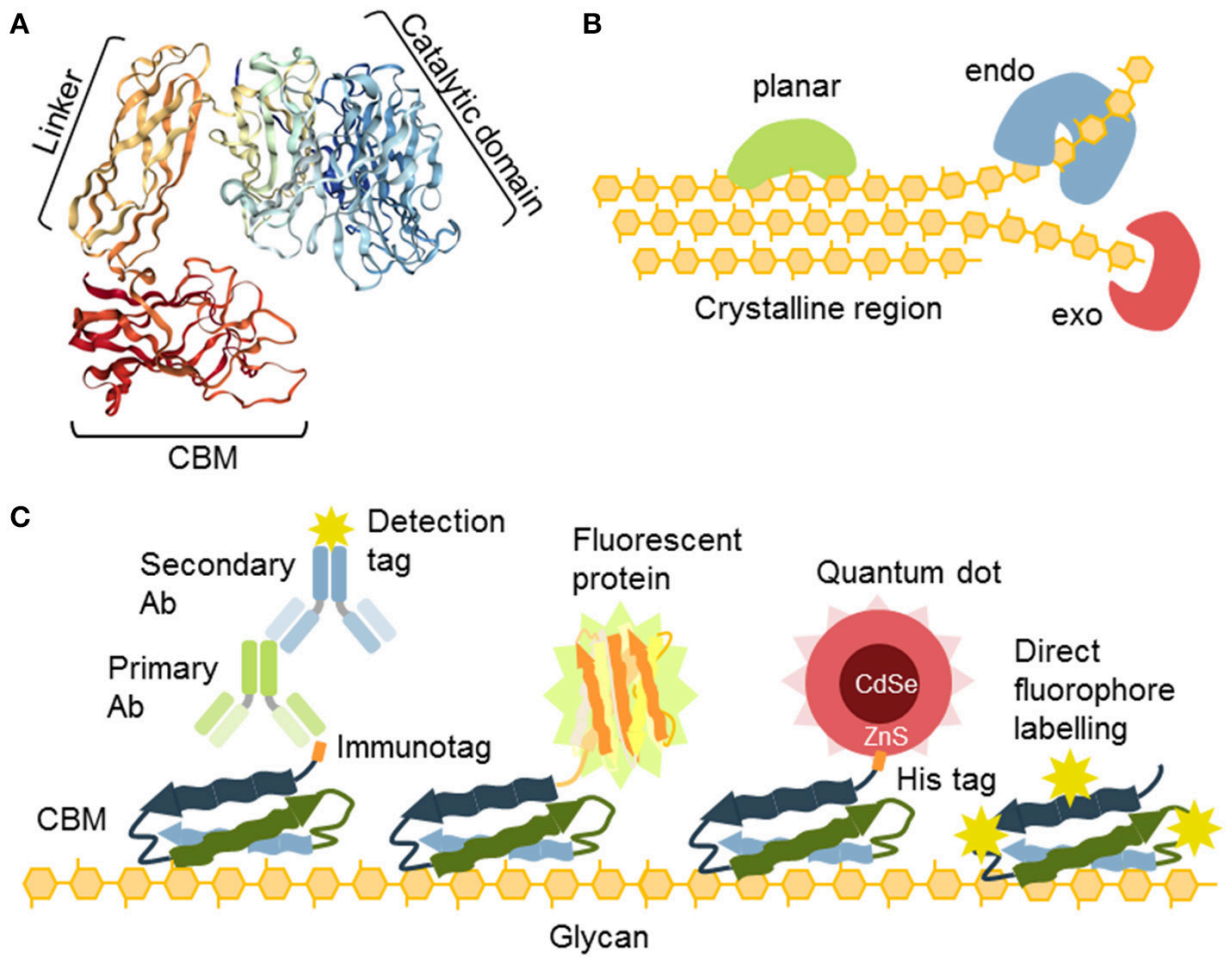
The nature and versatility of CBMs as probes. **(A)** CBMs are often a part of glycosyl hydrolases connected to the catalytic unit with a flexible linker. **(B)** Different types of cellulose-specific CBMs. Some binding is planar, such as to the crystalline part of cellulose. Some require amorphous parts and their binding can be characterized as “endo” and some bind ends of chains and can be characterized as “exo” binders. **(C)** CBMs offer a variety of secondary detection methods: (i) detection via immunotags (His, GST), (ii) as fusion constructs with fluorescent proteins, (iii) through coordination binding of the His tag with ZnS shell of quantum dots, (iv) CBMs can also be directly conjugated to fluorophores.

Some proteins, or parts of proteins, exhibit an affinity for carbohydrates as an essential feature of their activity. As such, they can be also used as probes (Figure 4; Table 6). For instance, carbohydrate-active enzymes (CAZYmes)^2^ are frequently appended with non-catalytic carbohydrate-binding modules (CBMs), typically 5–20 kDa in size (Gunnarsson et al., 2004), through a highly flexible linker (Gilbert et al., 2013; Figure 4A). CBMs are, by the CAZy database^2^ (The CAZypedia Consortium, 2018) definition, a contiguous amino acid sequence within a carbohydrate-active enzyme with a discrete fold having carbohydrate-binding activity. In some cases, large multi-enzyme complexes known as cellulosomes, with several CBMs appended, have also been described (>3 MDa) (Fontes and Gilbert, 2010; Smith et al., 2017). Currently, known CBMs are grouped into 83 families based on their amino acid sequence^3^ These can further be sub-grouped into CBM A-, B-, or C-type depending on their binding properties to polysaccharides (Figure 4B). Type A, B, and C CBMs are defined as binding crystalline surfaces, glycan chains, and short oligosaccharide sequences respectively (Boraston et al., 2004). Through substrate targeting, CBMs have the ability to enhance the activity of appended enzymes (Hervé et al., 2010). Although the convergent binding specificity may assign a CBM to a certain family, the function of the specific structure might be divergent (Montanier et al., 2009).

**Table 6:** Examples of carbohydrate-binding modules.

**Molecular probe**	**Epitope**	**References**
CBM3a	Cellulose (crystalline)/xyloglucan	Blake et al., 2006; Hernandez-Gomez et al., 2015
CBM4-1	Cellulose (amorphous)	Blake et al., 2006
CBM4-2	Xylan	Simpson et al., 2002
CBM9-2	End of glucan chains	Boraston et al., 2001
CBM20	Starch/glycogen	Jiang et al., 2010
CBM27	Mannan	Boraston et al., 2003
CBM61	Galactan	Cid et al., 2010
CBM76	Xyloglucan	Venditto et al., 2016
CBM77	HG with a low DE	Venditto et al., 2016

Expressed separately, these modules can be utilized in the context of the CWP as recombinant proteins (Figure 4C). Their detection is accomplished via an immunological tag (His, myc, GST, or other), or they can be used directly as fusion proteins with fluorescent proteins (Porter et al., 2007; Kawakubo et al., 2010; Badruna et al., 2017) or as conjugates to fluorophores (Dagel et al., 2011; Ding et al., 2012; Khatri et al., 2016). Interestingly, His-tagged CBMs can also be detected using quantum dots (brightly fluorescent semiconductor particles) with an outer shell that contains ZnS, which allows direct coordination by binding directly to a His-tag (Ding et al., 2006; Figure 4C).

The possibility of the mutational manipulation of the protein sequences of CBMs generates a momentum for engineering new specificities, which has already been explored to some extent leading to the creation of xylan and xyloglucan-specific CBMs (Gunnarsson et al., 2006), amongst others. This was accomplished by generating a combinatorial library of CBMs using limited substitution of specific amino acids in the binding cleft of the module (Gunnarsson et al., 2004, 2006). Consequently, the potential for diversification of CBMs as molecular probes has been shown. A major challenge in the post-genomic era is the analysis of ligand specificity for predicted CBMs, derived from genomic sequencing. While there is a growing list of over 80.000 modules that are assigned as CBMs in the CAZy database, only a minute fraction of these have been studied in detail and empirically tested (Gilbert et al., 2013; Venditto et al., 2016), and only a handful are regularly used as probes.

### Labeled cell wall degrading enzymes as probes

Cell wall component-degrading enzymes usually exhibit high levels of substrate specificity (Obeng et al., 2017) and the 3D molecular structure of many enzymes, and their different functional domains, have been described. Site-directed mutation can inactivate catalytic sites, leaving the binding site intact and functional, creating a possibility for novel proteinaceous probes. This has, for example, been explored by Dornez et al. (2011), who created an arabinoxylan-specific probe using an inactivated and fluorophore-labeled xylanase from *Bacillus subtilis*. It has also been shown that labeled active enzymes, like cellulases, can be used, particularly to study different aspects of the spatiotemporal dynamics of cellulose degradation (Ding et al., 2012; Wang et al., 2012; Luterbacher et al., 2013). Although these proteinaceous probes have been shown to provide high levels of resolution and specificity (Ding et al., 2012; Wang et al., 2012), they are still waiting for a more widespread usage in fields outside of biomass biotechnology.

## Small molecular CWPs—a new set of fine tools

Many of the proteinaceous CWPs mentioned above are suitable for many applications but have serious limitations with respect to the tracking of polysaccharides in living systems, and in the real-time study of the dynamics of cell wall architecture. Small direct molecular probes can negate some of the shortcomings of typically much larger proteinaceous CWPs. Developments in this field have been very successful over the last decade with some innovative solutions.

### Fluoro(chromo)phores—the smallest specific binders available

One of the most classical cytological methods to detect cell wall components is the use of dyes and stains. Although these stains are useful for morphological studies (especially Toluidine Blue which is dichromatic), they usually do not provide sufficiently high-resolution images and their specificity is often questioned. Despite this caveat, some are strongly favored and are not yet replaceable. Such an example of this would be the Yariv reagent for diagnostic detection and purification of arabinogalactan proteins (AGPs), or phloroglucinol (Wiesner stain), an easy and inexpensive reagent to detect lignin. For an overview of cell wall cytological staining methods see Krishnamurthy (1999).

**Figure 5:**
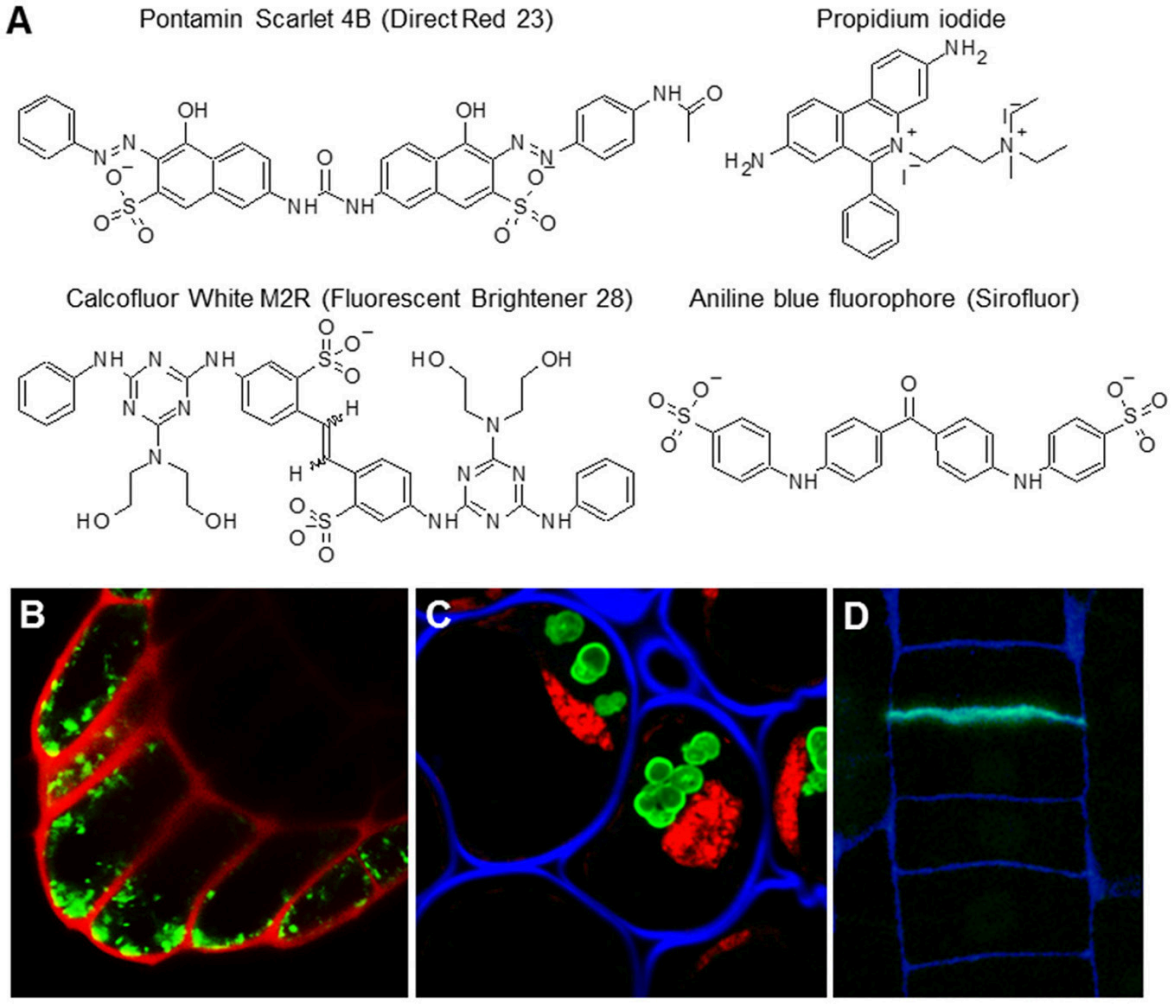
The most common cell wall-related fluorophores. **(A)** Molecular structures of four fluorophores. **(B)** Propidium iodide (PI) is traditionally used as cell wall counterstain for *in vivo* imaging of expression and localization of fluorescent proteins. Scan of the Arabidopsis columella root cap expressing YFP marker (green) counterstained with PI (red). **(C,D)** The fluorophores can be used in tandem and are compatible with other detection methods like immunolocalization. **(C)** Staining of the section of resin embedded pea root cap with INCh1 (anti-starch antibody, starch granules), Calcofluor White (blue; cell walls), and PI (red, staining nuclei). For the original experiments see Rydahl et al. (2017). **(D)** Sirofluor is specific for callose (Stone et al., 1982) which is present also in newly made cell walls. Example of a resin section of a root epidermis stained with Sirofluor (green) and Calcofluor White (blue). Note the septum-specific staining of Sirofluor.

Unlike classical stains for light microscopy, fluorophores, also called fluorochromes, or simply fluorescent dyes, can provide higher resolution images (Paës, 2014). Moreover, due to their low toxicity, they are sometimes suitable for *in vivo* imaging, even when using spinning disc or super-resolution confocal microscopy (Anderson et al., 2010; Liesche et al., 2013). They are relatively small but, although this is advantageous for *in vivo* studies, this feature can also significantly limit their specificity. The most commonly used fluorophores are listed in Table 7 and their structures and examples of their uses *in situ* are shown in Figure 5. Calcofluor White has been traditionally used as a general cell wall counterstain in immunohistological analyses. However, it is important to point out that it is not a cellulose dye as sometimes claimed—it binds all sorts of plant cell wall, bacterial, and fungal β-glucans including chitin. The advantages of Calcofluor White is its ease of visibility, rapid labeling, inexpensiveness, and its spectral properties that make it suitable for multichannel confocal imaging (Harrington and Hageage, 2003). Unlike Calcofluor White, some fluorochromes exhibit relatively high levels of specificity. Pontamine Scarlet 4B, for example, is more target-specific and its binding to cellulose can be successfully used for high-resolution *in vivo* studies (Anderson et al., 2010; Liesche et al., 2013), such as studying the deposition and orientation of cellulose microfibrils during cellular elongation (Anderson et al., 2010). Sirofluor is an essential fluorophore for *in situ* visualization of callose deposition (Evans and Hoyne, 1982; Stone et al., 1982). Propidium iodide (PI) has been used for a long time as a cell wall-illuminating counterstain for *in vivo* imaging of genetic markers with fluorescent proteins. One report more closely elaborated on PI binding and reported that PI competes with calcium in association with negatively charged pectins (Rounds et al., 2011). However, plant cell walls also contain other types of charged molecules and PI specificity has not been determined by more elaborate *in vitro* methods. Here it is important to note that PI staining of cell walls works only during *in vivo* imaging but not on fixed and sectioned material. PI exhibits a strong affinity for nucleic acids (this property of PI is used in other models), and prefers them over any cell wall material in fixed samples (Figure 5).

**Table 7:** Examples of fluorophores binding cell wall components.

**Molecular probe**	**Binding site**	**Spectral Properties**	**References**
Calcofluor White M2R (Fluorescent Brightener 28)	β-D-glucans (e.g. cellulose and chitin)	λex 347 nm/λem 430 nm	Harrington and Hageage, 2003
Aniline Blue Fluorophore (sirofluor)	(1 → 3)-β-D-glucan (callose)	λex 390 nm/λem 480 nm	Evans and Hoyne, 1982; Stone et al., 1982
Propidium iodide (PI)	Acidic polymers	λex 482 nm/λem 608 nm	Rounds et al., 2011
Pontamine Scarlet 4B (direct red23)	Cellulose microfibrils	λex 510 nm/λem 570 nm	Anderson et al., 2010; Liesche et al., 2013

### Oligosaccharide conjugates—emerging probes

**Figure 6:**
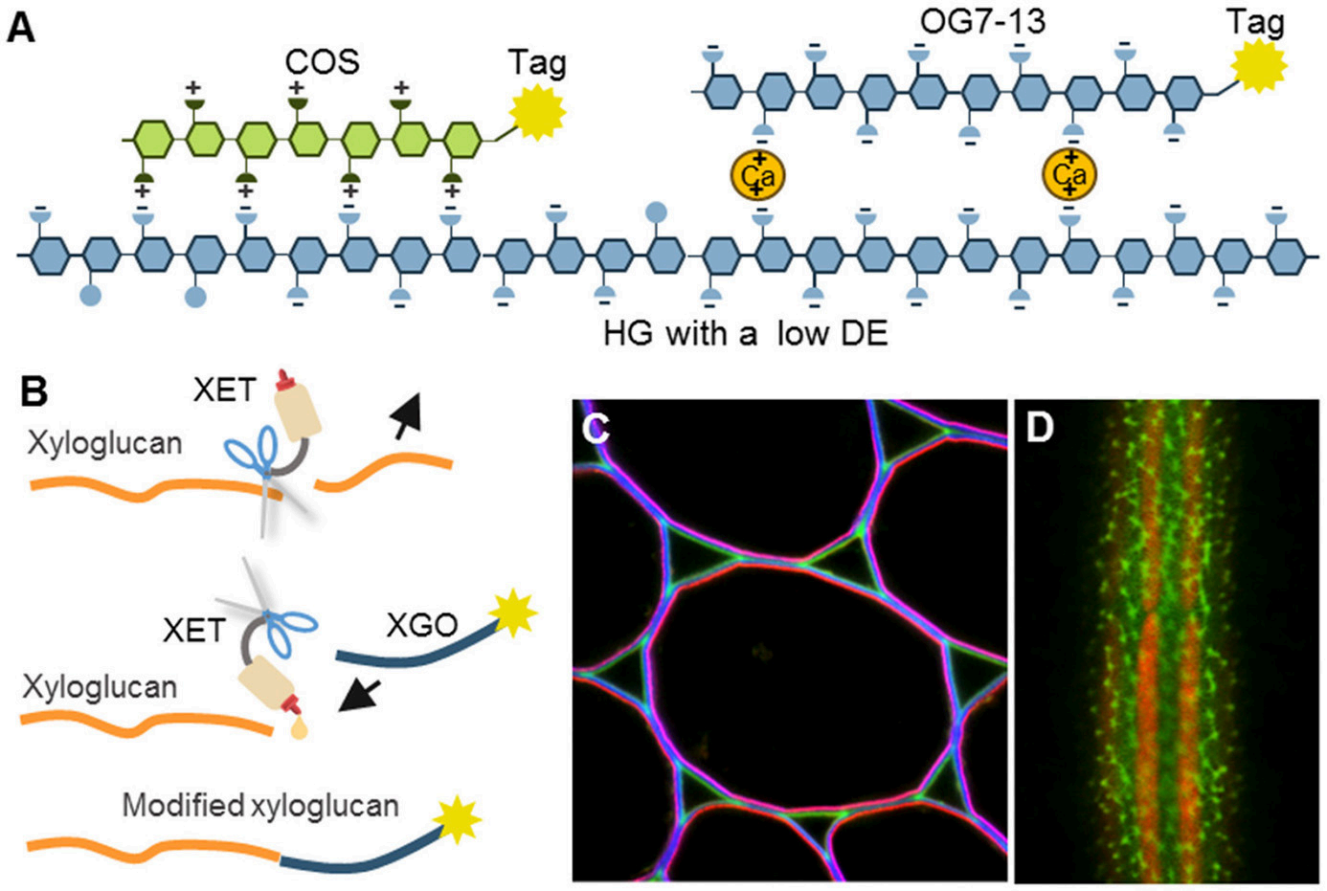
The mechanisms of oligosaccharide probes and examples of their usage. **(A)** Homogalacturonan (HG) with a low degree of esterification (DE) can be detected by positively charged chitosan oligosaccharides (COS) or by calcium-mediated complexation to long oligogalacturonides (OG7-13) as a form of artificial egg box formation (Mravec et al., 2014, 2017b). **(B)** Incorporation of tagged xyloglucan (XGO) oligosaccharides to xyloglucan backbone by an activity of xyloglucan endotransglycosylases (XETs). This can be used to visualize the presence of both, a xyloglucan backbone and XET activity. **(C,D)** Two examples of *in situ* usage of COS oligosaccharide probes. **(C)** Triple labeling of stem parenchyma with COS^488^ (green), Calcofluor White (blue) and JIM7 antibody (red). Note the specific labeling of the middle lamella and triangular junctions with COS^488^. **(D)** Labeling of intricate cell wall structures in single cell green alga *Penium margaritaceum* with COS^488^ (green). The red signal is due to chlorophyll autofluorescence. For the original experiments see Mravec et al. (2014).

Interestingly, certain oligosaccharides can also be used as CWPs (Table 8; Figure 6). For example, it has been known for a long time that defined fragments of xyloglucan (XGO) are substrates for “cut and paste” enzymes – xyoglucan endotransglycosylases (XETs) (Franková and Fry, 2013). When tagged XGOs are applied to plants their incorporation localizes the XET activity *in vivo* but also demonstrates the presence of the acceptor—the xyloglucan backbone (Vissenberg et al., 2000, 2001; Figure 6B). Particular physicochemical properties of oligosaccharides can be exploited as well. Recently, we introduced the chitosan oligosaccharide (COS) and oligogalacturonide (OG)-based probes (Mravec et al., 2014, 2017b; Figure 6A). Chitosan is a de-acetylated form of chitin and it bears positive charges in a spatial configuration that can interact with carboxyl groups on HG lacking methyl esters. Through a strong ionic interaction, the long OGs have been shown to be able to generate complexes with endogenous homogalacturonan in the presence of divalent cations, and can therefore mark sites with the ability for *de novo* egg box-formation (Mravec et al., 2017b; Figure 6A). This approach has demonstrated the dynamics of pectin complexation in tip growing structures: pollen tubes and root hairs. It is important to note that both probes can also be elicitors of cellular responses; chitin is a pathogen associated molecular pattern (PAMP) while OGs are damage-associated molecular patterns (DAMPs) (Malinovsky et al., 2014). Despite this disadvantage, they provide versatility in terms of different tags due to the possibility of conjugation to various florescent tags and even nanogold particles (Mravec et al., 2014). They are smaller than antibodies and exhibit a better penetration capacity than mAbs (Mravec et al., 2014). Unfortunately, these types of probes are currently limited to certain polysaccharides with special physicochemical properties. However, the association of two oligo(poly)saccharides can actually be governed by other molecular interactions, including hydrogen bonding or van der Waals forces. This means that structural modeling in combination with chemical synthesis of diverse artificial oligosaccharides might in theory expand this section of the toolbox in the future.

**Table 8:** Examples of oligosaccharide-based probes.

**Molecular probe**	**Binding site**	**References**
Chitosan oligosaccharide (COS)	HG with a low DE	Mravec et al., 2014
Oligogalacturonide (OG), DP7-13	HG with a low DE capable of *de novo* formation of egg boxes	Mravec et al., 2017b
XGO, DP 7-9 (XLLG, XXLG, XXXG)	Acceptor xyloglucan backbone	Vissenberg et al., 2000, 2001

### New toolbox compartments filled with the metabolic labeling-ready reagents

**Figure 7:**
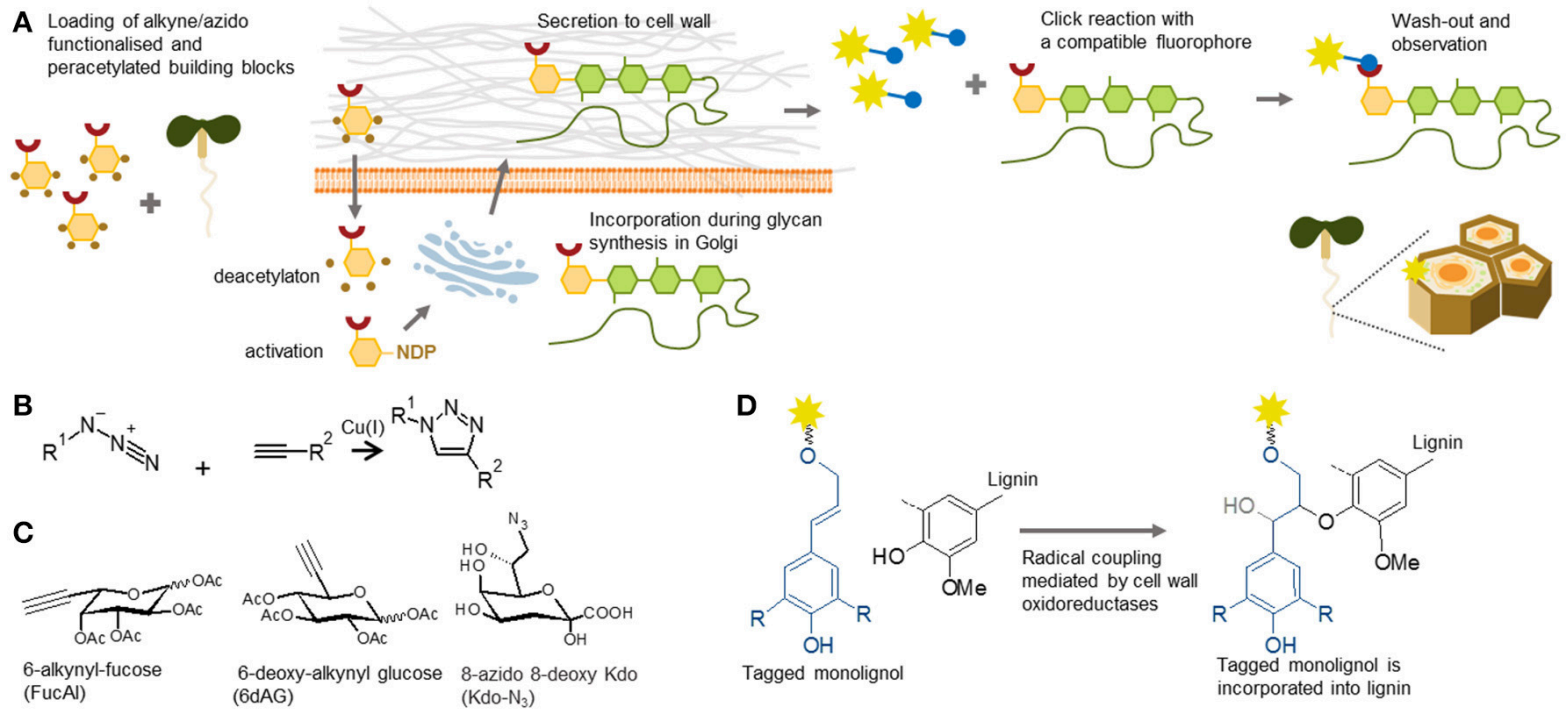
Metabolic labeling. **(A)** Principle of metabolic labeling or click chemistry-based tracking of polysaccharides in plants. Functionalized monosaccharides are loaded into plants. After entering the cell, they are incorporated by biosynthetic pathways pertinent to the respective glycan. The tracking analysis is enabled by a click reaction to a fluorophore containing a compatible reactive group. **(B)** Reaction scheme of Huisgen cycloaddition used in click chemistry. The reaction is catalyzed by Cu(I) and requires alkyne and azide groups, one present on the target molecule and one on the detection tag. **(C)** Three examples of click-chemistry-ready sugar analogs used in the cell wall field: 6-alkynyl-fucose (Anderson et al., 2012), 6-deoxy-alkynyl glucose (McClosky et al., 2016) and 8-azido 8-deoxy Kdo (Dumont et al., 2016). **(D)** Proposed mechanism of incorporation of functionalizes, in this case fluorophore-tagged monolignols, into lignin according Tobimatsu et al. (2013).

Tagging of cell wall components by so-called metabolic labeling involves the incorporation of the fluorescent analog/conjugate of a tagged building block into a native molecule *in planta* and subsequent tracking, a technique which is becoming increasingly important (Figure 7). The introduction of click chemistry technology to cell biology has been hugely effective, and facilitated the tracking of molecules in living models. Click chemistry is a term that was coined by Prof. Sharpless in the early 90's (Kolb et al., 2001). By this definition, the reaction can be called “click” when it is highly specific and efficient, and can be performed under mild conditions. For *in situ* probing, the most commonly used reaction is the Huisgen bipolar cycloaddition—a reaction between alkyne and azide in the presence of monovalent copper as a catalyst (Rostovtsev et al., 2002; Figure 7B). The ingenious idea behind the method is that one of these biorthogonal (biologically inert) groups can be part of a building block or a molecule of interest, and the other one can be the detection tag, usually a fluorophore. It is important to bear in mind that the copper catalyst is toxic for plants. However, this can be overcome by using a copper-free system, such as strained alkynes (Baskin et al., 2007), which can be used for tracking in live cells.

### Metabolic reagents for polysaccharides and proteoglycans

Click chemistry has numerous applications in glycobiology studies of animal and yeast systems (Yoon et al., 2017). In 2012, this method was also introduced into the field of plant cell wall biology (Anderson et al., 2012). An alkyne-containing derivative of fucose (FucAl), previously used in animal glycosylation, has been shown to be incorporated into pectin chains (as part of RG-I), which has enabled visualization of the arrangement and sites of pectin delivery at the plasma membrane-cell wall interface (Anderson et al., 2012). A dramatic increase in the panel of available click chemistry-enabled monomers has been reported in the past 2 years and now encompasses almost the whole spectrum of monosaccharides. For example, it now includes azido-KDO, which is incorporated into RG-II chains (Dumont et al., 2016), and alkyne-glucose (McClosky et al., 2016), which is incorporated into a non-specified component of the root hair cell walls and arrests their growth (Figure 7C). These constructs were recently followed by many more, including those that can be used to study *O*- and *N*-glycosylation of cell wall proteoglycans (Hoogenboom et al., 2016; Zhu and Chen, 2017). Although these monosaccharide analogs have been evaluated *in situ*, and some clearly generate cell wall staining, the precise targets have not yet been elucidated by other genetic, physiological, or analytical methods. We consider this a major challenge for the future work within the field of plant polysaccharide and proteoglycan metabolic labeling.

### Metabolic labeling reagents for lignin

The expansion of metabolic labeling tools can be also seen in the area of lignin biology. The first such reagents reported, were direct conjugates of fluorophores to monolignols (three types of hydroxycinnamyl alcohols). In glycan structures a large fluorophore molecule on the monomer would likely hinder the activity of the necessary glycosyltransferase as it would not be recognized as an appropriate natural donor. However, lignin polymerization is based on radical coupling and fluorescently labeled monolignols have been shown to be efficiently incorporated into lignin in stem cells undergoing formation of secondary cell walls (Tobimatsu et al., 2011, 2013; Figure 7D). Moreover, these reagents even enabled visualization of local deposition of polyphenolics within the Casparian strip of root endodermis (Tobimatsu et al., 2013). In addition to these fluorescent monolignols, several different click chemistry-ready monolignols, have been synthesized recently (Bukowski et al., 2014; Tobimatsu et al., 2014; Pandey et al., 2015). The latest publication (Lion et al., 2017) provided the most striking demonstration, how a variety of click chemistry reagents can be used for *in situ* studies of tissue specific lignification and formation of distinct types of lignin. The utilization of two types of clickable monolignols, H unit tagged with an azide group and G unit tagged with an alkyne group in tandem, enabled researchers to follow the dynamics of lignification in flax stems. We believe that by a similar approach, many of the less understood phenomena, such as turnover and salvage pathways of cell wall components (Barnes and Anderson, 2017), can be deciphered using either glycan or polyphenolic metabolic labeling.

### Conclusions and future directions

Recent progress in plant glycobiology, chemical biology, and HTP analytical methods has expanded the probing tool box plant researchers can use in their studies. However, the development of new CWPs is still progressing, and will undoubtedly mimic the great boost in biomedical probing technologies that we have seen in the past 20 years. We expect that the antibody field will benefit from the recently explored approach of a more biology- than researcher-driven selection process, shifting the current paradigm of anti-glycan antibody production requiring predefined targets. The more widespread use of synthetic antibodies, using recombinant methods and expression, might pave the way for complex targets to be addressed, such as those for which the deconstruction of their native 3D context is an issue in conventional procedures. This is especially relevant for non-covalent interlinks within cell walls. The large collection of mAbs available, as well as CBMs, offer a hitherto unexplored resource for generating genetic probes. In this approach, the DNA coding sequences of the known proteinaceous binders are expressed *in planta* via stable transgenic constructs as fusion proteins with a small immunotag or fluorescent protein. Although this approach might be problematic for visualization of epitopes in the context of the whole organism, or tissues, due to the constant and ubiquitous expression, it might be useful for studying the dynamics of cell wall microdomains. It is well-known that the binding of an antibody can influence the antigen's expected biological activity so the genetic approach can also provide entirely new tools for functional analyses of cell wall components. Proteinaceous probes can be further engineered by random or via site-directed mutagenesis to widen the diversity of specificities or to modulate their affinity.

Other tools that have already found important uses in biomedical research, like aptamer and affimer technologies, can also be applied to cell wall components, thus opening new frontiers for replenishing the shortage of probes against supramolecular conformations, intermolecular linkages, and fine 3D arrays of polysaccharides *in muro*. Aptamers are DNA/RNA oligonucleotides (Zhang et al., 2016) while affimers are small proteins (Tiede et al., 2017) exhibiting levels of affinity and specificity similar to mAbs. Due to their small-size, they can be beneficial in applications where accessibility of the epitope is an issue. Furthermore, since they can be developed against a target in its native state, aptamers also offer the possibility for quantification using standard molecular biology methods like qPCR. Interestingly, several anti-glycan DNA aptamers have already been generated, such as those against cellulose (Yang et al., 1998). However, they have not yet been fully investigated as CWPs. The future work will certainly also involve further development of the HTP methods to analyze the specificity and sensitivity of the generated CWPs. HTP profiling platforms, especially those involving synthesized defined oligosaccharides, are a relatively recent advance and their potential has not been fully explored. The combination of HTP screening with large chemical libraries and combinatorial synthesis of fluorophores or artificial oligosaccharides can be another valuable source of exciting new tools for the cell wall probing toolbox.

## Author contributions

MR: drafted the part dealing with mAbs and CBMs; AH: drafted the part about phage display; SK and JM: drafted the parts dealing with small molecular probes while JM assembled the figures, and performed the compilation and editing.

### Conflict of interest statement

The authors declare that the research was conducted in the absence of any commercial or financial relationships that could be construed as a potential conflict of interest.

## Footnotes

^1^http://glycomics.ccrc.uga.edu/wall2/antibodies/antibodyHome.html

^2^http://www.cazy.org

^3^http://www.cazy.org/Carbohydrate-Binding-Modules.html.

## Abbreviations

AGP: arabinogalactan protein
AP: alkaline phosphatase
CBM: carbohydrate-binding module
CDTA: cyclohexanediaminetetraacetic acid
CoMPP: comprehensive microarray polymer profiling
COS: chitosan oligosaccharide
DE: degree of esterification
DP: degree of polymerisation
HG: homogalacturonan
HRP: horse radish peroxidase
HTP: high throughput
KLH: keyhole limpet hemocyanin
KDO: 3-Deoxy-D-*manno*-oct-2-ulosonic acid
NDP: nucleotide diphosphate
OG: oligogalacturonide
PI: propidium iodide
PS4B: Pontamine Scarlet 4B
RG-I/-II: rhamnogalacturonan I/II
XET: xyloglucan endotransglycosylases
XGO: xyloglucan oligosaccharide.
